# Innovative Methods Used in Monitoring COVID-19 in Europe: A Multinational Study

**DOI:** 10.3390/ijerph20010564

**Published:** 2022-12-29

**Authors:** Brigid Unim, Nienke Schutte, Martin Thissen, Luigi Palmieri

**Affiliations:** 1 Department of Cardiovascular, Endocrine-Metabolic Diseases and Aging, National Institute of Health, Via Giano della Bella 34, 00162 Rome, Italy; 2 Department of Epidemiology and Public Health, Sciensano, Rue Juliette Wytsmanstraat 14, 1050 Brussels, Belgium; 3 Unit 24—Health Reporting, Department of Epidemiology and Health Monitoring, Robert Koch Institute, General-Pape-Str. 62-66, 12101 Berlin, Germany

**Keywords:** COVID-19, coronavirus, pandemic, digital tools, mobile devices, health monitoring

## Abstract

Several innovative methods have been deployed worldwide to curb the COVID-19 pandemic. The aim of the study is to investigate which innovative methods are used to monitor COVID-19 health issues in Europe and related legislative and ethical aspects. An online questionnaire was administered to European countries’ representatives of the project Population Health Information Research Infrastructure. Additional information was obtained from websites and documents provided by the respondents; an overview of the literature was also performed. Respondents from 14 countries participated in the study. Digital tools are used to monitor the spread of COVID-19 (13/14 countries) and vaccination coverage (12/14); for research, diagnostics, telehealth (14/14); to fight disinformation (11/14) and forecast the pandemic spread (4/14). The level of implementation of telehealth applications was mostly low/medium. Legislative and ethical issues were encountered in many countries, leading to institutional distrust. The COVID-19 pandemic has highlighted the need for timely and accurate health data for research purposes and policy planning. However, the use of innovative methods for population health monitoring and timely data collection has posed challenges to privacy and online security globally. Adequate regulatory oversight, targeted public health interventions, and fight against disinformation could improve the uptake rate and enhance countries’ emergency preparedness.

## 1. Introduction

As recently demonstrated by the COVID-19 pandemic, health is a cross-border issue that requires coordinated global efforts to collect and share information between countries for research purposes and policy making [1]. During the coronavirus pandemic, several innovative solutions have been developed worldwide to monitor the spread of the coronavirus, research and develop diagnostics, vaccines and teleconsultations, and to fight disinformation. Innovative solutions include, but are not limited to, national contact tracing and warning applications (apps) or digital tools used to notify users, even across borders, if they have been in contact with an infected person. These are usually installed into mobile phones and exchange information with other mobile phones in proximity to trace potentially infected individuals. In the case of an alert, the app may provide relevant information from health authorities, such as advice to get tested or to self-isolate, and information about COVID-19 contact centers [2]. Many information communication technologies-ICTs (e.g., Bluetooth, Global Positioning System—GPS, WiFi, QR codes) have been adopted to enable contact tracing via digital tools [3]. 

Innovative solutions are also applications using artificial intelligence (AI) to detect patterns in the spread of the coronavirus, facilitating COVID-19 public health monitoring within a community and rapid response strategies. Other digital solutions and tools that have been developed across countries to monitor the spread of the coronavirus, often without a specific law, are eBracelets, smart cameras providing facial recognition, thermal scans, drones, robot surveillance, and websites with health questionnaires where symptoms can be reported [4]. Online platforms fighting disinformation are another example of innovative methods used in the containment of the pandemic. In 2020, the European Commission (EC) highlighted the importance in tackling COVID-19 disinformation, thus false or misleading information (misinformation) about coronavirus. Therefore, a program to monitor the actions of online platforms to limit the spread of COVID-19 disinformation, especially on social media (e.g., Google, Facebook, Twitter, and Microsoft) [5] was developed by the EC. 

The EC also funded several projects to ’advance the understanding of the novel coronavirus epidemic, contribute to more efficient clinical management of patients infected with the virus, as well as public health preparedness and response’ [6]. Among these, the Population Health Information Research Infrastructure (PHIRI) project [1] aims to facilitate and support research activities in Europe through the sharing of COVID-19 population health information and exchange of best practices related to the identification of data sources, access, and reuse of COVID-19 data according to Ethical, Legal and Social Implications (ELSI) and FAIR Data principles (Findable, Accessible, Interoperable, and Reusable) [7]. These goals will be achieved in 36 months (November 2020–November 2023) through the collaboration of 41 partners across 30 countries. In line with the project’s objectives, the present study aims to investigate which innovative methods, state-of-the-art algorithms and digital tools (i.e., social media platforms, contact tracing devices, and AI applications) are being used across Europe to monitor health issues related to COVID-19, as well as who is using them. Key considerations on the role of legislative and ethical aspects are also examined to provide relevant recommendations, facilitating innovation uptake and diffusion.

## 2. Materials and Methods

Data collection for the present study was performed using three methods: online survey, web-based search and overview of the literature.

### 2.1. Online Survey

A questionnaire (Supplementary Material File S1) was designed from October to November 2021. The starting point in the development of the survey tool was the EC website providing the definition of innovative solutions and examples of digital tools used during the coronavirus crisis. The digital tools were categorized in three groups: (1) national contact tracing and warning apps; (2) artificial intelligence; and (3) online platforms fighting disinformation [2]. The survey questions were initially based on these three categories and were further expanded to include teleconsultations/telemedicine, as well as legislative and ethical aspects. An additional question was added to capture any other innovative solution used in Member States but not mentioned in the questionnaire. The questionnaire was reviewed by the research team with the addition of questions about end users, uptake rates and impact evaluation of the digital tools. The survey instrument was then piloted among five researchers and minor edits were made for clarity.

The final version of the questionnaire was administered online from December 2021 to April 2022 through Microsoft Forms, that stores data on servers in Europe. The survey addressed National Nodes (NN) representatives who are researchers, epidemiologists, and policy makers affiliated with European health and research institutions, including universities. A National Node is an organisational entity linked to a national institution or governmental unit that brings together national stakeholders in a country (e.g., national statistical office, national public health institute, representatives from ministries of health, education and research) [8]. As partners of the PHIRI project, the NN representatives provided their contact details to the project consortium and agreed to submit COVID-19 relevant information when required. The contact list of the consortium was used to recruit the NN representatives who were asked to share the questionnaire with their colleagues involved in COVID-19 surveillance and monitoring at regional or national level. To enhance the response rate, two reminder emails were sent in January and March 2022. The survey period was also extended for a month, and then closed at the end of April 2022.

The questionnaire was composed of three questions on socio-demographic factors (name, country of origin, and email contact) and 27 questions related to innovative tools, distributed in four sections:

(i) Innovative solutions implemented in the country. This section included questions on digital solutions used to: (a) monitor the spread of the coronavirus; (b) research and develop diagnostics and telemedicine; (c) monitor COVID-19 vaccine uptake; and (d) fight disinformation. Responders were asked to provide, for each tool, information about guidelines or best practices, uptake rate, target population, and impact assessment. Concerning disinformation, respondents were asked to provide the names and/or web links of existing platforms;
(ii) Algorithms. This section inquired about the availability of specific algorithms (AI) to detect patterns in the spread of the coronavirus (e.g., supercomputers). The names and/or web links of available documents were required;
(iii) Comments. Respondents could provide further information about the implementation of innovative solutions in their country;
(iv) Legislative and ethical aspects. The section included a question on legislative and ethical aspects concerning the use of digital tools addressing the COVID-19 pandemic. Respondents were also asked to provide the title and/or web links of available documents.

A descriptive analysis was performed using the statistical package SPSS v.28 (IBM SPSS Statistics for Windows, Armonk, NY, USA: IBM Corp).

### 2.2. Web-Based Search

Information about digital tools implemented in the countries of the survey participants were completed using publicly available data from the EC website [9] and websites provided by the respondents. Each website was searched for relevant information about general characteristics (i.e., name of the tool, main functions, target population, uptake rate), technical aspects (i.e., type of server and communication technology, data on impact assessment, activity status), and ethical and legal issues related to digital tools. The retrieved information was stored in an excel spreadsheet and used for a descriptive summary.

### 2.3. Literature Review

An overview of the literature was also performed when information was not available or accessible from the aforementioned sources. The search on Pubmed was conducted using combinations of the following search term: ‘(COVID-19 OR SARS-CoV-2) AND (digital tools OR digital devices OR mobile devices OR online platforms OR digital platforms) AND (monitoring OR diagnosis OR telemedicine OR telehealth) AND (country)’. Relevant information regarding digital tools (i.e., general characteristics, technical, legal and ethical issues) was extracted from the 18 documents retrieved through the literature review and the 12 documents indicated by the respondents and saved in a excel spreadsheet. A summary description of the content of these documents was performed.

## 3. Results

The survey participants were 19 representatives from 14 European countries (Table 1); six countries (Croatia, Finland, Germany, Ireland, Italy and Spain) had two participants in the study. 

Innovative solutions implemented in Europe:

(i) Digital tools for COVID-19 monitoring.

Digital tools (e.g., national contact tracing and warning apps) are used to monitor the spread of the coronavirus in all countries, except in Serbia (Table 1). The main functions of the tools are contact tracing and COVID-19 symptoms checking or general health functionalities. Some digital tools, e.g., the National Health Service (NHS) COVID-19 in the United Kingdom (UK) and Zostan Zdravy in Slovakia, also offer the possibility to book for COVID-19 testing. The main users of the tools are the general population, healthcare providers, and epidemiologists. The uptake rate, in terms of downloads, varies across countries, ranging from 6% (Croatia) to 57% (Finland) of the population. It should be noted that the Slovak app was available for few months in 2020 as it was withdrawn by the Slovak National Health Information Centre due to privacy and security issues. The app was downloaded by almost 2% of the population during its first month of availability. Data on active users were reported for three countries (Finland, Ireland, and the Netherlands); the highest uptake rate of 100% was indicated for the COVID-19 surveillance tool adopted by all regional health services in the Netherlands. 

Out of 13 countries using digital tools for COVID-19 monitoring, 11 used a decentralized contact tracing system for data linkage and follow-up of the contacts (Table 2). A centralized contact tracing system was in place in Lithuania and Slovakia. Data exchange between mobile devices was performed via Bluetooth for all apps; GPS was also deployed in the Slovakian app for geolocalization. 

Impact assessment to evaluate the public health effectiveness of digital contact tracing tools was reported for eight countries (Croatia, Finland, Germany, Italy, the Netherlands, Portugal, Spain, and the UK). The assessments consisted of population-based surveys, pilot studies, evaluations performed by expert groups providing technical reports, in-depth analysis of COVID-related data and comments from the end users. At the time of writing this article, six countries (Austria, Finland, Ireland, The Netherlands, Portugal, and Slovakia) have discontinued their contact tracing apps. Further information, including guidelines or examples of best practices, on digital monitoring are accessible on the websites provided for each tool.

(ii) Digital tools used for research and development of diagnostics and telemedicine.

Digital tools used for research, diagnostics and telemedicine are currently available in all countries considered in the study, although with variable implementation level (Table 3). The tools included the Central Patient Data Register, telemedicine applications, online conference platforms, and various digital health apps for chronic patients or the general population. Guidelines or examples of best practices related to the tools are available in most countries. However, the documents are not publicly accessible in Slovakia. The target groups of these tools were the general population, patients, and also healthcare providers using them for virtual visits, patient remote monitoring and management. The tools were also deployed for consultations with other specialists, referrals or transitions of care. Other users were epidemiologist accessing clinical data for research activities. The highest implementation rate was observed in Finland, Portugal, Slovakia, and Slovenia for digital services used in all health facilities at regional or national level. Impact assessment of the digital devices performed by government authorities are available for Germany, Ireland, Italy, Portugal, and Spain. 

The results of the literature review indicated a non-optimal uptake of telemedicine in Austria, Germany, the Netherlands and the UK. However, the adoption rates increased during the COVID-19 pandemic [10,11,12,13]. A major uptake in the German medical practice was observed for telemedicine and teletherapy applications during the pandemic; contrarily for telemonitoring devices for chronic patients [14]. In the UK, the higher levels of remote consultations were observed in primary care, and mostly consisted in telephone consultations and text messaging, while video consultations were used to a lesser extent [15]. Similar findings were observed in Austria [11]. On the contrary, video consultations increased in primary care services in the Netherlands during the pandemic, while text messaging and telephone consultations were already diffuse before the coronavirus outbreak [13].

The literature studies regarding Finland depicted a strong infrastructure for eHealth services that currently connects all healthcare service providers. Telemedicine and digital apps are highly diffuse and facilitate access to health services and patient management. Finland has two decades of experience in telemedicine that was further expanded during the pandemic. Currently, over 50% of the Finnish population and almost 40% of healthcare professionals use virtual care [16,17]. Lithuania introduced remote consultations in primary care during the pandemic. The new model of healthcare service delivery required changes to the legal framework, as well as a rapid development of online platforms and contact centers to inform the general population and support primary healthcare. A year after the coronavirus outbreak, remote consultations became routine practice in primary healthcare in Lithuania [18]. As in Lithuania, telemedicine was not widely implemented nor regulated in Serbia prior to the pandemic. Several telemedicine pilot projects have been launched in August, 2022. In particular, the pilot project on telephone consultations in primary care is expected to start soon [19].

(iii) COVID-19 vaccine monitoring tools.

National online platforms (dashboards) are publicly available, except in Serbia and Portugal, for the monitoring of COVID-19 vaccination coverage. They usually provide up-to-date information about vaccination uptake by region, type of vaccine, number of vaccine doses administered, and age groups. These data are used to issue COVID-19 vaccine certificates or pass.

Electronic vaccination registries are also used to monitor COVID-19 vaccination uptake (the Netherlands, Slovenia) and, besides health professionals, registered citizens can access their data. In Spain, a computerized database of primary care medical records (the BIFAP database), owned by the Spanish Agency for Medicines and Medical Devices, collects data about vaccinated individuals and adverse events. The data is used for conducting pharmaco-epidemiological studies; access is granted to health professionals and researchers. In Italy, an immunization register was implemented at national level by the Ministry of Health two years prior to the coronavirus outbreak. The register collects data from all regions to monitor the immunisation coverage, including COVID-19 vaccination. Researchers from Italian national institutes were granted access to COVID-19 surveillance data, including the immunisation register, during the emergency through the Ordinance of 27 February 2020, n. 640 issued by the Head of the Civil Protection Department [20].

(iv) Online platforms fighting disinformation.

Online platforms against disinformation on COVID-19 or screening for products (e.g., food) with alleged healing or protective effects are available in 11 out of 14 countries in the study (Table 4). 

These platforms include official websites of Ministries of Health (Italy, Portugal), National Institutes of Public Health (Croatia, Italy, The Netherlands, Slovenia), health services providers (Ireland), websites supported by the government (Germany, Slovakia, the UK) and national associations (Austria, Italy). Disinformation about coronavirus is also addressed through social media accounts (e.g., Facebook, TikTok, Twitter, Instagram) of national institutes and through search engines (Google). 

### 3.1. Algorithms (Artificial Intelligence) Used in Detecting the Spread of Coronavirus

According to the respondents, specific algorithms capable of detecting patterns in the spread of the coronavirus (e.g., supercomputers) are available in four countries (Germany, Italy, Spain, UK). The German ‘Corona-Datenspende’ predicts SARS-CoV-2 infections based on data from wearables fitness devices with inbuilt sensors (e.g., accelerometers, temperature and optical sensors). The sensors collect health data, which are then forward as anonymized data packages to a server. Information from several sensors are combined and analysed to create a Fever Map to detect regions or hot spots in which the number of residents with fever symptoms is higher than the average [21]. Another example from Germany is the project ‘Control and prognosis of intensive care COVID-19 capacities (SPoCK)’. The project provides forecasts of the expected number of COVID-19 patients requiring intensive care. The data used for modeling are prevalence and incidence rates of COVID-19 infections and the capacities of intensive care units. In this way, the project supports hospitals in the management and transfer planning of COVID-19 patients [22].

Italy is currently leading the project ‘Exscalate4CoV’ that developed a powerful and cost-efficient intelligent supercomputing platform to identify the molecules capable of targeting the coronavirus and to design an effective tool to contain future pandemics [23].

In Spain, AI devices are used to detect COVID-19 clusters [24], forecast excess mortality or all-cause mortality related to surface temperature [25], and to identify the main factors influencing the uneven spread of SARS-CoV-2 in Spain. These factors are mobility of the population (which directly implies social contact), percentage of infected health personnel and the place of residence for every 100 individuals over 70 years old [26].

The ‘QCOVID’ tool containing algorithms to identify patients at high risk of severe COVID-19 outcomes have been used in the UK. Moreover, two new risk prediction algorithms have been validated to estimate the risk of COVID-19 related mortality and hospital admission in vaccinated individuals [27].

Specific algorithms are not available in Austria, Croatia, the Netherlands, and Serbia, while respondents from six countries were not aware of the existence of such algorithms in their countries (Finland, Ireland, Lithuania, Portugal, Slovakia, Slovenia). 

### 3.2. Other Types of Innovative Solutions 

The respondents provided information about other innovative solutions available in their countries. Croatia developed a digital assistant ‘Andrija’, in collaboration with epidemiologists, to advice the general population during the pandemic. Andrija is a ‘virtual doctor’ using AI to assist the population in diagnosing and managing of COVID-19 infections. Andrija can also connect citizens with health authorities for further information and assistance in order to prevent health system overload. Croatia has also developed the ‘COVIDGO’ mobile app to verify and save the EU digital COVID-19 certificate. Similar apps were reported for other countries (Ireland, Lithuania, Italy).

Online platforms for reporting and support of victims of domestic violence during the pandemic isolation were mentioned by respondents from Croatia and Germany. A platform to seek financial aid during the emergency is also available in Germany. Several online platforms were used across Europe for distance teaching and learning during lockdowns (e.g., Teams, YouTube, gSuite for Education, Google classroom, Zoom).

### 3.3. Legislative and Ethical Aspects

The survey participants provided examples of specific legal measures introduced by their government to support surveillance and contain the pandemic using digital solutions, and to facilitate COVID-19 data collection and sharing. This was accompanied with the creation or reorganization of specific committees advising the government and bringing together experts from different fields of work (epidemiology, bioethics, ICTs, etc.). 

The EC issued several recommendations to establish common rules in using mobile applications and health data for modeling or forecasting the spread of COVID-19 [28,29]. The European Data Protection Board also deals with data protection issues and developed guidelines on the use of location data and contact tracing tools during the coronavirus outbreak [30]. These regulations, guidelines and recommendations were adopted by European Union Member States (EU MS). Indeed, data protection authorities were involved in the development of the apps across Europe, and a data controller responsible for the app’s data processing, was nominated in each country. An exception was Slovenia, where the Information Commissioner was not involved in the development process of the COVID-19 monitoring app and a data controller was not appointed by the Slovenian government [31]. In Slovakia, the government approved the law ‘Lex Corona’ allowing the National Health Authority (NHA) to request telecommunication providers to provide data from mobile devices to enable contact tracing. After the adoption of the law, the Constitutional Court of the Slovak Republic suspended the effect of the law due to risk of limitation of human rights and freedom. Although the Parliament amended the law, obliging the NHA to adopt organizational and technical measures for data and privacy protection, the app Zostan Zdravy was discontinued shortly after [31].

Recommendations of the World Health Organization (WHO) regarding certification of death during the pandemic were also adopted (e.g., Italy, Spain) to guarantee high quality of information in the medical death certificate [32].

## 4. Discussion

Many countries have adopted digital technologies to monitor the spread of the coronavirus and to stop or limit disease transmission. Innovative solutions also included digital devices for educational purposes, telehealth, platforms to curb disinformation and prevent home violence. The least adopted tools were AI-based devices that can be used to identify, track, and forecast outbreaks and diagnose the virus.

Most of these technologies (e.g., contact tracing apps) involve personal data processing, and therefore require the application of privacy protection laws. At the beginning of the pandemic, the EC issued several recommendations for the adoption of these technologies while the responsibility of their implementation relied on the EU MS. Notwithstanding, data breaches and privacy issues have been registered in several countries, leading to legal amendments, modifications or discontinuity of the digital tools. For instance, several regional web and mobile apps were introduced in Spain and many of them violated the individual’s rights to privacy and security. Although the national app Radar COVID was released after a successful testing phase, the data protection impact assessment and the source code were not released on time. Another example is the NHS COVID-19 app, which was based, at first, on a centralized data collection system and was therefore criticized due to possible privacy breaches. In response, the NHS turned to the decentralized system [31]. Similar issues have been observed in Europe and beyond, declining public trust in COVID-19 measures [33,34]. These events were exacerbated by the attempts of some governments to render COVID-19 apps mandatory, as witnessed in Austria, Portugal, and Slovenia [31]. Although the use of innovative technological solutions was necessary to curb the pandemic, citizens’ fundamental rights and freedoms should not be jeopardized even in times of emergency. The implementation of new technologies according to the data protection law in the EU—the General Data Protection Regulation (GDPR)—could increase the uptake rate and strengthen health systems, ensuring an adequate response to future health threats.

Institutional distrust has serious consequences. First of all, non-adherence to public health interventions to mitigate the spread of coronavirus, such as vaccine hesitancy. Secondly, lack of perceived susceptibility or severity to the disease and low health literacy, in addition to institutional distrust, could lead to misinformation, conspiracy theories or disinformation. In this light, ensuring that trusted and reliable institutions provide public health information is of paramount importance. The documentations regarding public health measures should be accurate, in plain language, and easily accessible. An effective dissemination of health information could be achieved by considering the intended target groups and choosing the appropriate reporting format [35]. In addition, public health interventions to improve general health and vaccine literacy, especially among vulnerable populations (e.g., the elderly, migrants, the homeless) should be a political priority [36,37].

According to our findings, AI-based devices capable of predicting the spread of the pandemic were the least used tools. Although a wide adoption of these technologies by health systems could enhance classic public health measures to curb the pandemic, challenges related to the availability of in-house expertise in specific disciplines, quality of the data used, and incorporation of epidemiological features need to be approached. Indeed, few European countries have the ability to perform foresight studies using AI (e.g., Spain, Germany, the UK) which are necessary to better understand future health impacts of the SARS-CoV-2 pandemic and for policy planning [38]. Improving data collection and curation, promoting sharing of data and source codes across countries, training activities, interdisciplinary teams of modelers from the fields of infectious diseases and clinical epidemiology could enhance the prediction of epidemic models and the implementation level [39].

The pandemic has certainly impacted healthcare systems globally, leading to new patterns in medical practices to maintain physical distancing and reduce overload of national health systems. Consequently, telemedical applications were widely adopted. Telemedicine, or telehealth, is defined as the use of medical data that is exchanged through ICTs for medical purposes [40]. There are many applications of telehealth technologies, ranging from real-time video consultations with various specialists (e.g., cardiologists, oncologists, dermatologists, psychiatrists); telephone, e-mail, and video services in primary care for counseling and management of patients with chronic or other conditions; to hospital-based services (e.g., intensive care, emergency and trauma care, stroke intervention) [41]. Privacy and data security are also important in this context to ensure informed consent of the patients, secure data transmission and to enable the participation of service providers who are not subject to professional secrecy [40]. ICT training programs for healthcare professionals are recommended to limit the underutilization of new technologies for medical purposes. 

One of the lessons learned from the pandemic is the urgent need for well established ICT infrastructures at national levels. The countries with good level of adoption of digital technologies in the healthcare system (e.g., Finland, Slovenia) were advantaged during the health crisis that required real-time public health surveillance, high quality health data from multiple data sources, interoperability of ICT systems for data linkage, management of big data, and availability of qualified personnel. Countries with medium/low level of health digitalization had more challenges. For instance, Italy struggled during the early stages of the pandemic due to limited secondary use of data caused by strict legislation, strenuous bureaucratic data access processes, and long recruitment process of professional figures. In addition, limited infodemic management and communication gaps with the general population at regional and national level led to the low adoption rate of the national contact tracing device ‘Immuni’. However, Italy benefited from the well established vaccination information system implemented before the COVID-19 crisis that uses the same surveillance system in all regions, allowing for real-time data collection, sharing and analysis of vaccination data. These data flows are based on emergency legislations and more structural responses are required for long term effects beyond the crisis [42]. 

A limitation of the present study is the small sample size. However, this is a qualitative study and information provided by the respondents, including documents and websites, were sufficient to depict the degree of implementation of digital tools in their countries. A literature overview was also performed to complete information for some countries. Another limitation is the language barrier encountered in the study as information was available only in national language on some websites and documents indicated by the responders. The contents of these references were translated into English using Google translate or by native speakers. Furthermore, other aspects accelerated by digital technologies, and their societal impact, were not explored in the current study. It should be noted that new technologies have improved and accelerated various processes, such as vaccine development and drug discovery. The global response to COVID-19 has shown that vaccine development can be accelerated without compromising on safety. Other vaccines, such as those against malaria and tuberculosis, might benefit from this experience. Future studies could investigate other methods and uses of new technologies to better understand the direct and indirect effects of health digitalization. 

## 5. Conclusions

In conclusion, the pandemic has highlighted the need for timely and up-to-date health data for research and national policy planning. Although innovative methods for public health monitoring positively impacted healthcare systems across Europe, privacy and security issues were the major barrier to the implementation of the new technologies. Adequate performance oversight of the wide range of digital tools is required to guarantee the rights and freedom of all citizens even in times of global emergencies. Additionally, targeted public health interventions to improve health literacy, ICT training courses addressing health professionals, major transparency in the development of the digital tools, as well as improved data and source code sharing could increase the implementation level of digital solutions, curb misinformation and disinformation, and strengthen emergency preparedness in Europe.

## Figures and Tables

**Table 1:** General characteristics of digital tools used for COVID-19 monitoring in Europe.

**Country**	**Digital Tool**	Function	Target Population	Uptake Rate (As of April, 2022)		Website
				% Active users	Downloads (% of Population)	
Austria	STOPP CORONA	contact tracing; health functionalities	general population	nr	1.4 million (16%, February 2021)	https://www.roteskreuz.at/site/meet-the-stopp-corona-app/ (accessed on 16 May 2022).
Croatia	Stop COVID-19	contact tracing	general population	nr	236,553 (6%)	https://www.koronavirus.hr/stop-COVID-19/723 (accessed on 16 May 2022).
Finland	Koronavilkku (CoronaBlinker)	contact tracing	general population	nr	2.75 million (50%)	https://koronavilkku.fi/en/ (accessed on 17 May 2022).
	OMAOLO	COVID-19 symptoms checking	general population	1.72 million (30%, January 2021)	na	https://www.omaolo.fi/ (accessed on 17 May 2022).
Germany	Corona-Warn-App	contact tracing	general population, health care providers	nr	over 47 million (57%)	https://www.coronawarn.app/en/ (accessed on 17 May 2022); https://www.coronawarn.app/en/analysis/ (accessed on 17 May 2022).
Ireland	COVID Tracker	contact tracing; COVID-19 symptoms checking	general population	1.7 million (35%)	nr	https://www2.hse.ie/services/COVID-tracker-app/why-use-the-COVID-tracker-app.html (accessed on 19 May 2022).
Italy	IMMUNI	contact tracing	general population	nr	21.8 million (37%)	https://www.immuni.italia.it/ (accessed on 19 May 2022).
Lithuania	Korona STOP LT	contact tracing; health functionalities	general population	nr	350,000 (12.5%)	https://koronastop.lrv.lt/en/ (accessed on 18 May 2022).
The Netherlands	CoronaMelder	contact tracing	general population	over 2 million (12%)	nr	https://coronamelder.nl/en/ (accessed on 18 May 2022).
	Clusterbuster	regional COVID-19 surveillance (clusters, outbreaks, vaccination rate)	public health physicians, epidemiologists	100%	na	https://www.rivm.nl/regionale-infectieziektebestrijding/clusterbuster-regionale-surveillance-applicatie-COVID-19 (accessed on 20 May 2022); https://www.rstudio.com/blog/how-the-clusterbuster-shiny-app-helps-battle-COVID-19-in-the-netherlands/ (accessed on 20 May 2022).
Portugal	Stayaway COVID App	contact tracing	general population, primary care physicians, chronic patients	nr	2.9 million (25%, January. 2021)	https://stayawayCOVID.pt/en/ (accessed on 20 May 2022).
Serbia	No monitoring app	na	na	na	na	na
Slovakia	Zostan Zdravy (Stay Healthy)	contact tracing; testing	general population, health care providers, epidemiologists	nr	Over 90,000 (2%, April 2020)	https://github.com/ct-report/SK (accessed on 24 May 2022).
Slovenia	OstaniZdrav (Stay Healthy)	contact tracing	general population	nr	over 460,000 (22%)	https://www.gov.si/en/topics/coronavirus-disease-COVID-19/the-ostanizdrav-mobile-application/ (accessed on 24 May 2022).
Spain	Radar COVID	contact tracing; health functionalities	general population	nr	nr	https://radarCOVID.gob.es/en/terms-and-conditions-use (accessed on 25 May 2022); https://radarCOVID.gob.es/recursos-de-comunicacion (accessed on 25 May 2022).
United Kingdom	NHS COVID-19 app	contact tracing; COVID-19 symptoms checking, testing	general population	nr	29.5 million (50%)	https://COVID19.nhs.uk/ (accessed on 26 May 2022).

nr: not reported; na: not applicable; NHS: National Health Service.

**Table 2:** Technical aspects and current status of digital tools used for COVID-19 monitoring in Europe.

Country	Digital Tool	Centralized or Decentralized Server	Communication Technology	Impact Assessment (Public Health Effectiveness)	Status (as of September 2022)
Austria	STOPP CORONA	decentralized	Bluetooth	No	Discontinued from 28 February 2022
Croatia	Stop COVID-19	decentralized	Bluetooth	Yes (end users)	Active
Finland	Koronavilkku (CoronaBlinker)	decentralized	Bluetooth	No	Discontinued
	OMAOLO	decentralized	web-based	Yes (survey)	Active
Germany	Corona-Warn-App	decentralized	Bluetooth	Yes (app analytics, survey)	Active
Ireland	COVID Tracker	decentralized	Bluetooth	No	Discontinued for contact tracing
Italy	IMMUNI	decentralized	Bluetooth	Yes (surveys, reviews, evaluation team)	Active
Lithuania	Korona STOP LT	centralized	Bluetooth	No	Active
The Netherlands	CoronaMelder	decentralized	Bluetooth	Yes (evaluation team)	Temporarily suspended from 22 April 2022
	Clusterbuster	nr	nr	Yes (end users)	Active
Portugal	Stayaway COVID App	decentralized	Bluetooth	Yes (evaluation team)	Discontinued
Serbia	No monitoring app	na	na	na	na
Slovakia	Zostan Zdravy (Stay Healthy)	centralized	Bluetooth, GPS	No	Discontinued
Slovenia	OstaniZdrav (Stay Healthy)	decentralized	Bluetooth	No	Active
Spain	Radar COVID	decentralized	Bluetooth	Yes (pilot study)	Active
United Kingdom	NHS COVID-19 app	decentralized	Bluetooth	Yes (surveys)	Active

nr: not reported; na: not applicable; NHS: National Health Service.

**Table 3:** Digital tools used for research and development of diagnostics and telehealth.

Country	Digital Tool	Implementation Level	Impact Assessment of the Tool	Guidelines or Best Practices
Austria	Telemedicine, telephone and e-consultations	Low/medium	na	na
Croatia *	Telemedicine (teleradiology)	Low	No	✓
Finland	Telemedicine, digital health devices	High	Yes	✓
Germany	Telemedicine (teletherapy), telemonitoring devices	Low/medium	Yes (German Digital Healthcare Association)	✓
Ireland	Telemedicine	na	Yes (HSE National Telehealth Steering Committee)	✓
Italy	Telemedicine, digital health devices	Low/medium	Yes (Italian Digital Health Observatory)	✓
Lithuania	Telemedicine	Low/medium	na	na
The Netherlands	Telemedicine, telephone and e-consultations	Low/medium	na	na
Portugal	Telemedicine	High	Yes (system analytics)	✓
Serbia	Telemedicine	Low	na	na
Slovakia	Digital health devices	High	No	✓
Slovenia	Central Patient Data Register, Telemedicine, online conference platforms	High (Central Patient Data Register)	No	✓
Spain **	Telemedicine, digital health devices	Low/medium	Yes (Spanish Agency on HTA)	✓
United Kingdom	Telemedicine, telephone and e-consultations	Medium	na	na

HSE: Health Service Executive; HTA: Health Technology Assessment; na: not available. * https://www.hzhm.hr/en/telemedicine/teleradiology (accessed on 16 June 2022); ** https://cms.law/en/int/expert-guides/cms-expert-guide-to-digital-health-apps-and-telemedicine/spain (accessed on 23 June 2022); https://eithealth.eu/in-your-region/spain/ (accessed on 23 June 2022).

**Table 4:** Online platforms fighting COVID-19 related disinformation.

Country	Platforms Against Disinformation	Name of the Platforms	Web Link
Austria	✓	Austrian Health Literacy Alliance	https://oepgk.at/fake-news/ (accessed on 8 July 2022). https://oepgk.at/english-summary/ (accessed on 8 July 2022).
Croatia	✓	Croatian Institute of Public Health	https://www.hzjz.hr (accessed on 8 July 2022).
Finland	__	__	__
Germany	✓	Facts for Friends	https://factsforfriends.de/about-us (accessed on 12 July 2022).
Ireland	✓	HSE through Facebook and Instagram	ns
Italy	✓	VaccinarSi	https://www.vaccinarsi.org/ (accessed on 12 July 2022).
		Ministry of Health	https://www.salute.gov.it/portale/nuovocoronavirus/archivioFakeNewsNuovoCoronavirus.jsp (accessed on 12 July 2022).
		National Institute of Health	https://www.iss.it/en/primo-piano/-/asset_publisher/3f4alMwzN1Z7/content/COVID-dall-iss-un-vademecum-contro-le-fake-news-sui-vaccini (accessed on 15 July 2022).
		Facebook	https://it-it.facebook.com/formedia/tools/coronavirus-resources (accessed on 15 July 2022).
Lithuania	__	__	__
The Netherlands	✓	RIVM controls information on social media	https://www.rivm.nl/en (accessed on 18 July 2022).
Portugal	✓	Ministry of Health	https://COVID19.min-saude.pt/ (accessed on 18 July 2022).
Serbia	__	__	__
Slovakia	✓	Government website: Coronavirus (COVID-19) in the Slovak Republic	www.korona.gov.sk (accessed on 18 July 2022).
Slovenia	✓	National Institute of Public Health through its social media accounts (Facebook, TikTok, Instagram, Twitter)	https://www.facebook.com/nijz.si (accessed on 22 July 2022); https://www.tiktok.com/@_nijz (accessed on 22 July 2022); https://www.instagram.com/_nijz_/ (accessed on 22 July 2022); https://twitter.com/NIJZ_pr (accessed on 22 July 2022).
		Cepimose (Let us vaccinate)	www.cepimose.si (accessed on 26 July 2022).
Spain	✓	Maldita	https://maldita.es/ (accessed on 26 July 2022).
		Law against disinformation (2020)	https://www.boe.es/eli/es/o/2020/10/30/pcm1030 (accessed on 26 July 2022).
United Kingdom	✓	UK Government through Facebook, Twitter, TikTok, Google, Apple News	https://www.ofcom.org.uk/research-and-data/media-literacy-research/coronavirus-resources (accessed on 26 July 2022).

HSE: Health Service Executive; RIVM: National Institute for Public Health and the Environment; ns: not specified; __: no data.

## Data Availability

All data generated or analyzed during this study are included in this article.

## Supplementary Materials

The following supporting information can be downloaded at: https://www.mdpi.com/article/10.3390/ijerph20010564/s1, Supplementary Material File S1: Survey on innovative methods for health monitoring in Europe: Digital solutions addressing the COVID-19 pandemic.
