# Supplementary material for: Innovative Methods Used in Monitoring COVID-19 in Europe: A Multinational Study
Source: Int J Environ Res Public Health. 2022 Dec 29;20(1):564. doi: 10.3390/ijerph20010564 (PMC9819661; doi:10.3390/ijerph20010564)
Supplement: Supplementary file 1 [file ijerph-20-00564-s001.zip › ijerph-2002081-supplementary.pdf]

SURVEY ON INNOVATIVE METHODS FOR HEALTH MONITORING IN EUROPE

Digital solutions addressing the covid-19 pandemic

Questionnaire

1. Please, enter your first and last name ________________________________

2. Select your country

	Austria		Greece		Portugal
	Belgium		Hungary		Romania
	Bosnia and Herzegovina		Ireland		Serbia
	Bulgaria		Italy		Slovakia
	Croatia		Latvia		Slovenia
	Cyprus		Lithuania		Spain
	Czech Republic		Luxembourg		Spain
	Denmark		Malta		Sweden
	Estonia		Moldova (Republic of)		Switzerland
	Finland		Netherlands		United Kingdom
	France		Norway		
	Germany		Poland		

3. Enter your e-mail address ____

This project has received funding from the European Union's Horizon 2020 research and innovation programme under grant agreement No 101018317

Innovative solutions and digital tools implemented in your country

1. Which digital solutions addressing the covid-19 pandemic have been implemented in your country? Please, provide information on all digital solutions implemented in your country.

   A. Digital tools used to monitor the spread of the coronavirus (e.g., national contact tracing and warning apps)

- If guidelines or best practices on digital tools are available in your country, please list the documents and/or links related to the tool(s): ____
- What is the uptake rate of the tool(s)? ____
- Have measures been adopted to evaluate the impact of the digital tool(s) (e.g., user surveys)? If 'yes', please specify the measures____
- Which are the target groups of the tool(s) (e.g., general population, healthcare workers, patients)?____

   B. Digital tools used to research and develop diagnostics and teleconsultations

- If guidelines or best practices on digital tools are available in your country, please list the documents and/or links related to the tool(s): ____
- What is the uptake rate of the tool? ____
- Have measures been adopted to evaluate the impact of the digital tool(s) (e.g., user surveys)? If 'yes', please specify the measures____
- Which are the target groups of the tool(s) (e.g., general population, healthcare workers, patients)? ____

   C. Digital tools/ online platforms used to monitor vaccination coverage levels/vaccine uptake

- If guidelines or best practices on digital tools are available in your country, please list documents and/or links related to the tool(s): ____
- What is the uptake rate of the tool? ____
- Have measures been adopted to evaluate the impact of the digital tool(s) (e.g., user surveys)? If 'yes', please specify the measures____
- Which are the target groups of the tool(s) (e.g., general population, healthcare workers, patients)? ____

   D. Online platforms fighting disinformation, screening for food and non-food products with alleged healing or protective effects related to the coronavirus (e.g., Ebay, Facebook, Google, Microsoft)

   Name of the platforms and/or links ____

This project has received funding from the European Union's Horizon 2020 research and innovation programme under grant agreement No 101018317

Algorithms (artificial intelligence)

1. Are specific algorithms (artificial intelligence) available in your country to detect patterns in the spread of the coronavirus (e.g., supercomputers)? If ‘yes’, please provide the title of the document(s) and/or link.

   □ Yes ____

   □ No

   □ I don't know/not aware

Legislative and ethical aspects

1. Are there information on legislative and ethical aspects related to the use of digital solutions addressing the covid-19 pandemic in your country (e.g., guidelines, reports)? If ‘yes’, please provide the title of the document(s) and/or link.

   □ Yes ____

   □ No

   □ I don't know/not aware

Comments

1. Do you have further information or comments about the implementation of digital tools and innovative solutions in your country?____

This project has received funding from the European Union's Horizon 2020 research and innovation programme under grant agreement No 101018317
